# Beyond Terminator Narratives: Implantable Cardioverter-Defibrillators as a Lens for Clinical Agentic Artificial Intelligence

**DOI:** 10.1016/j.mcpdig.2026.100376

**Published:** 2026-05-23

**Authors:** Shreyas A. Modi

**Affiliations:** Division of Cardiology, Department of Medicine, VA Loma Linda Healthcare System, Loma Linda, California; Division of Cardiology, Loma Linda University, Loma Linda, California; Department of Internal Medicine, University of California, Riverside, California

Artificial intelligence (AI) in health care is rapidly evolving from passive decision support toward systems capable of monitoring clinical data streams and initiating actions within defined clinical workflows. These emerging systems are often described as agentic because they interpret signals and act toward predefined goals with limited real-time clinician input. Despite their promise, clinician trust in such systems remains cautious, reflecting concerns regarding reliability, interpretability, accountability, and workflow integration.^1^

Medicine has already incorporated a successful model of supervised autonomy in routine practice. Implantable cardioverter-defibrillators (ICDs) continuously monitor cardiac rhythm and deliver therapy automatically when programmed detection criteria are met.^2^^,^^3^ Their acceptance provides a useful lens for understanding how agentic clinical AI may be safely introduced into health care environments. The clinical acceptance of ICD therapy did not depend on eliminating uncertainty in arrhythmia detection but on embedding autonomy within explicit safety boundaries. Detection zones are programmable, therapies are tiered, clinician override remains available, and device behavior is continuously auditable through interrogation. These characteristics transformed what might otherwise have been perceived as uncontrolled automation into a trusted extension of physician intent.

The regulatory and clinical governance frameworks that supported ICD deployment could also offer guidance for oversight of emerging agentic AI systems. Device therapy succeeded because responsibilities were clearly distributed among manufacturers, clinicians, and health care systems. Programming parameters defined operational limits, postimplant monitoring ensured ongoing performance assessment, and adverse events remained traceable and reviewable through structured surveillance mechanisms. Similar lifecycle principles are reflected in regulatory approaches to software as a medical device, including the US Food and Drug Administration’s AI/machine learning–based software as a medical device action plan, which emphasizes risk-based evaluation, transparency, and real-world performance monitoring as essential components of safe clinical implementation.^4^

Large randomized clinical trials have traditionally served as the evidentiary foundation for introducing new therapies into medical practice. In contrast, many clinical AI systems are introduced following retrospective validation studies and staged implementation evaluations rather than randomized trials demonstrating improvements in clinical outcomes, making continuous postdeployment monitoring particularly important as these systems interact with evolving clinical environments.^1^^,^^5^ Recognizing this need, the American Heart Association has proposed a lifecycle framework for clinical AI spanning predeployment evaluation, implementation-phase oversight, and postdeployment monitoring, emphasizing that safety and reliability must be continuously reassessed as models are deployed across patient populations and clinical workflows. The advisory also provides four pragmatic guiding principles for health systems that are beginning to set up AI governance processes, including strategic alignment, ethical evaluation, usefulness and effectiveness evaluation, and financial performance, to inform health system selection, validation, deployment, and actionable monitoring of AI tools.^6^ Agentic AI systems will require comparable governance structures. Algorithms must operate within clearly specified clinical scopes, monitoring systems must detect performance drift over time, and institutions must define escalation pathways when unexpected outputs occur. Most importantly, continuous postdeployment monitoring is essential when agentic systems rely on complex or partially opaque model architectures whose internal decision pathways may not be directly interpretable by clinicians.

Artificial intelligence is increasingly transforming how medicine is practiced and delivered across clinical workflows, diagnostics, and decision-making.^6^ As agentic systems become increasingly capable of monitoring clinical data streams and initiating actions within defined clinical contexts, important questions arise regarding safety, accountability, and their impact on patients and the health care workforce. However, medicine has previously incorporated technologies that introduced new forms of supervised therapeutic autonomy, such as ICDs. Applying similar governance principles—supported by emerging lifecycle evaluation frameworks for clinical AI—may help ensure that agentic AI strengthens rather than disrupts clinical care. Moving beyond Terminator narratives requires recognizing that the central challenge is not autonomy itself, but how that autonomy is governed, monitored, and aligned with clinical responsibility as these systems enter routine clinical practice.

## Potential Competing Interests

The views expressed in this article are those of the author and do not necessarily reflect the position or policy of the U.S. Department of Veterans Affairs or the United States Government. The author reports no competing interests.

## Declaration of Generative AI and AI-Assisted Technologies in the Writing Process

During the preparation of this work, the author used ChatGPT and Claude in order to assist with formatting, language refinement, and references. After using this tool/service, the author reviewed and edited the content as needed and takes full responsibility for the content of the published article.
